# T315 Decreases Acute Myeloid Leukemia Cell Viability through a Combination of Apoptosis Induction and Autophagic Cell Death

**DOI:** 10.3390/ijms17081337

**Published:** 2016-08-15

**Authors:** Chang-Fang Chiu, Jing-Ru Weng, Appaso Jadhav, Chia-Yung Wu, Aaron M. Sargeant, Li-Yuan Bai

**Affiliations:** 1 Division of Hematology and Oncology, Department of Internal Medicine, China Medical University Hospital, Taichung 40447, Taiwan; d5686@mail.cmuh.org.tw; 2 Cancer Center, China Medical University Hospital, Taichung 40447, Taiwan; 3 College of Medicine, School of Medicine, China Medical University, Taichung 40402, Taiwan; 4 Department of Marine Biotechnology and Resources, National Sun Yat-sen University, Kaohsiung 80424, Taiwan; columnster@gmail.com; 5 Department of Biological Science and Technology, China Medical University, Taichung 40402, Taiwan; kelen0523@yahoo.com.tw; 6 Division of Medicinal Chemistry, College of Pharmacy, The Ohio State University, Columbus, OH 43210, USA; appaso06@gmail.com; 7 Charles River Laboratories, Preclinical Services, Spencerville, OH 45887, USA; aaron.Sargeant@crl.com

**Keywords:** T315, acute myeloid leukemia, apoptosis, autophagy, autophagic cell death

## Abstract

T315, an integrin-linked kinase (ILK) inhibitor, has been shown to suppress the proliferation of breast cancer, stomach cancer and chronic lymphocytic leukemia cells. Here we demonstrate that T315 decreases cell viability of acute myeloid leukemia (AML) cell lines (HL-60 and THP-1) and primary leukemia cells from AML patients in a dose-responsive manner. Normal human bone marrow cells are less sensitive than leukemia cells to T315. T315 down regulates protein kinase B (Akt) and p-Akt and induces caspase activation, poly-ADP-ribose polymerase (PARP) cleavage, apoptosis and autophagy through an ILK-independent manner. Interestingly, pretreatment with autophagy inhibitors rescues cells from apoptosis and concomitant PARP cleavage, which implicates a key role of autophagic cell death in T315-mediated cytotoxicity. T315 also demonstrates efficacy in vivo, suppressing the growth of THP-1 xenograft tumors in athymic nude mice when administered intraperitoneally. This study shows that autophagic cell death and apoptosis cooperatively contribute to the anticancer activity of T315 in AML cells. In conclusion, the complementary roles of apoptotic and autophagic cell death should be considered in the future assessment of the translational value of T315 in AML therapy.

## 1. Introduction

Acute myeloid leukemia (AML) is a hematological malignancy characterized by the proliferation of clonal neoplastic hematopoietic cells and diverse clinical presentations. Chemotherapy with or without hematopoietic stem cell transplantation remains the mainstay of AML treatment. While advances in medicine and supportive care have led to complete remission for 70%–80% of adult AML patients, only 20%–30% of these patients have long term disease-free survival [1]. The major cause of this discrepancy is the acquisition of chemoresistance in refractory or relapsed AML. Alternative compounds or strategies are therefore needed to more effectively manage patients with AML.

T315, *N*-methyl-3-(1-(4-(piperazin-1-yl)phenyl)-5-(4′-(trifluoromethyl)-[1,1′-biphenyl]-4-yl)-1*H*-pyrazol-3-yl)propanamide, was originally identified as an integrin-linked kinase (ILK) inhibitor and characterized by Lee et al. [2]. Subsequent studies demonstrate the efficacy of T315 against several types of cancers. In breast cancer, T315 suppressed γ-secretase-mediated Notch1 activation in caveolae of IL-6-abundant cells through inhibition of ILK [3]. Amelioration of NF-κB (nuclear factor κ-light-chain-enhancer of activated B cells) was thought to be responsible for the anticancer activity mediated by T315 in human gastric cancer cells [4]. T315 also has antitumor activity independent of the canonical ILK inhibition. In chronic lymphocytic leukemia, Liu et al. demonstrated that T315 directly abrogated protein kinase B (Akt) activation by preventing translocation of Akt into lipid rafts, and induced caspase-dependent apoptosis by suppressing B-cell receptor, CD49d, CD40, and Toll-like receptor 9-mediated Akt activation in an ILK-independent manner [5].

In the present study, we examine the anticancer activity and possible underlying mechanisms of T315 against two AML cell lines and primary leukemia cells from patients with AML. In addition, the ability of T315 to inhibit leukemia growth is demonstrated in athymic nude mice bearing THP-1 xenografts.

## 2. Results

### 2.1. T315 Increases Apoptotic Cells and Reduces Viability of Acute Myeloid Leukemia (AML) Cell Lines and Primary Leukemia Cells from AML Patients

**Figure 1:**
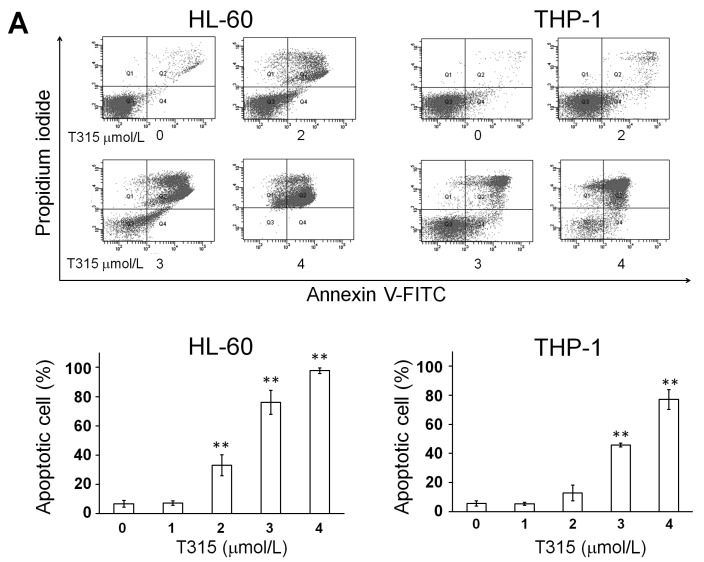

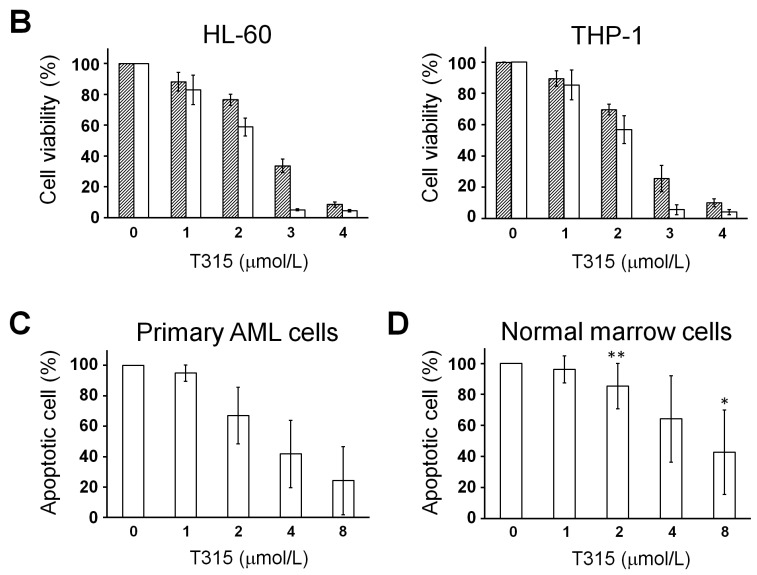
Cell viability inhibition study of T315 in acute myeloid leukemia (AML) cell lines, primary AML cells and normal marrow cells. (**A**) HL-60 and THP-1 cells (0.25 × 10^6^ cells/mL) were incubated with T315 or dimethyl sulfoxide (DMSO) vehicle for 24 h. The apoptotic cells were analyzed by annexin V-FITC and propidium iodide (PI) staining, as described in Materials and Methods. **Upper** panel: one example; **Lower** panel: apoptotic cell percentage (*n* = 3); (**B**) HL-60 and THP-1 cells (0.25 × 10^6^ cells/mL) were incubated with T315 or DMSO vehicle for 24 h (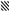) or 48 h (□). The cells were analyzed by MTS assay, as described in Materials and Methods; (**C**) Primary AML cells (0.25 × 10^6^ cells/mL) were incubated with T315 or DMSO for 24 h. The cells were stained with annexin V-FITC and PI to assess apoptotic cells percentage (*n* = 26); (**D**) Normal bone marrow nucleated cells (0.25 × 10^6^ cells/mL) were incubated with T315 or DMSO for 24 h. The cells were stained with annexin V-FITC and PI to assess apoptotic cells percentage (*n* = 16). * denotes *p* < 0.05; ** denotes *p* < 0.01 compared to the control group (in panel **A**) or compared to the primary AML cells at the same concentration of T315 (in panel **D**).

The annexin-V/PI staining and the MTS assay were used to determine the effect of T315 on the viability of HL-60 and THP-1 cells which were treated with 0, 1, 2, 3 or 4 µmol/L T315 for 24 or 48 h. There was a dose-dependent increase of apoptotic cells in both HL-60 and THP-1 cells treated with T315 (Figure 1A). Figure 1B demonstrates the dose and time-dependent decrease in cell viability induced by T315. The IC_50_ values were 2.53 and 2.72 µmol/L at 24 h, and 2.01 and 2.90 µmol/L at 48 h for HL-60 and THP-1, respectively.

In order to determine the efficacy of T315 on primary AML cell viability, freshly isolated AML cells were treated with T315 (ranging from 0, 1, 2, 4 and 8 µmol/L) and the cell viability was evaluated by annexin-V/PI staining analysis. The mean IC_50_ at 24 h for 26 patients was 4.2 ± 1.6 µmol/L (Figure 1C). Importantly, the normal bone marrow nucleated cells were less sensitive to T315 with an IC_50_ of 6 ± 1.9 µmol/L at 24 h (*n* = 16, Figure 1D). The IC_50_ of T315 for AML cells was significantly lower than the IC_50_ for normal marrow cells (*p* = 0.003).

### 2.2. T315 Induces Down-Regulation of Protein Kinase B (Akt) and Phosphorylated Akt in AML Cell Lines

**Figure 2:**
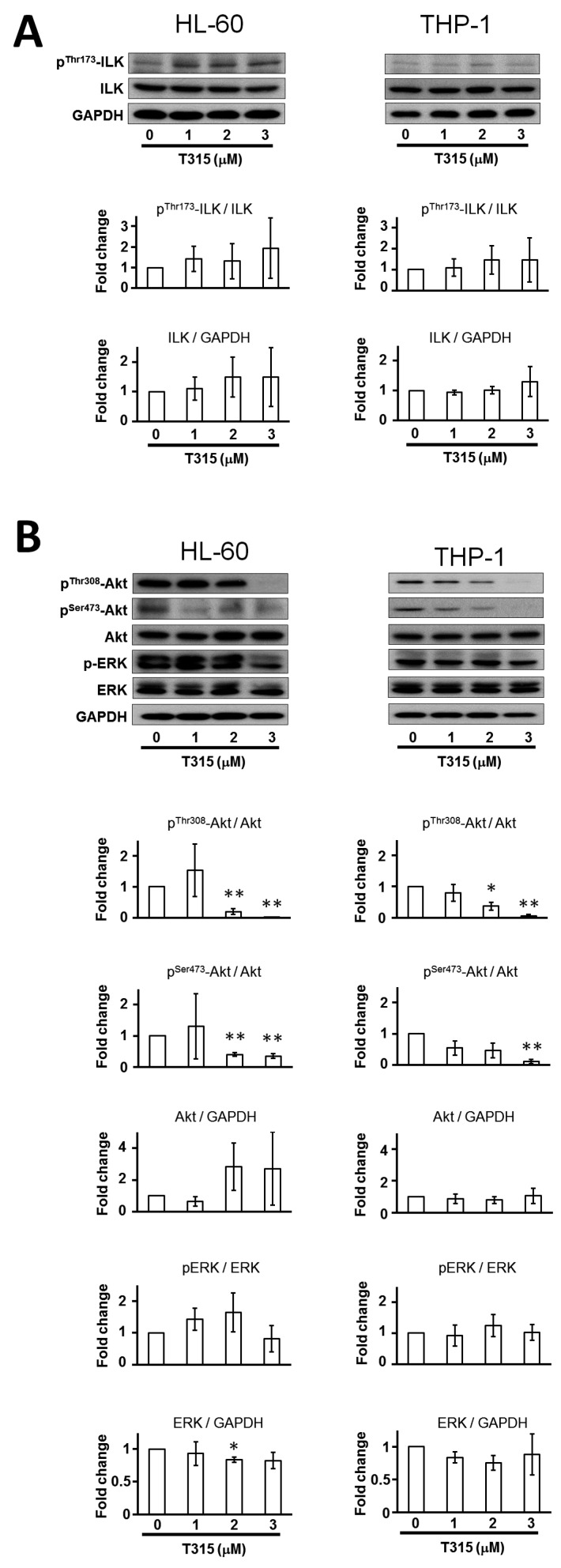
T315 induces dephosphorylation of protein kinase B (Akt) without change of integrin-linked kinase (ILK) in AML cell lines. Cells (0.25 × 10^6^ cells/mL) were treated with T315 at the indicated concentration or DMSO for 24 h, and 20 µg protein extract from cell lysates in each condition were used for Western blot analysis. (**A**) T315 did not change the p^Thr173^-ILK and total ILK expression. Histogram of fold change of p^Thr173^-ILK/ILK and ILK/glyceraldehyde 3-phosphate dehydrogenase (GAPDH) were shown in lower panels (*n* = 3); (**B**) T315 down regulated both p^Thr308^-Akt and p^Ser473^-Akt, but not Akt, p-ERK and ERK expression. Histogram of fold change of p^Thr308^-Akt/Akt, p^Ser473^-Akt/Akt, Akt/GAPDH, p-ERK/ERK, and ERK/GAPDH were shown in lower panels (*n* = 3). * denotes *p* < 0.05; ** denotes *p* < 0.01 compared to the control group.

T315 has been reported as an ILK inhibitor [2]. We evaluated the influence of T315 on the expression of p^Thr173^-ILK and total ILK, as well as proteins regulating cell proliferation and survival in AML cells (Figure 2). T315 treatment did not change the protein expression of p^Thr173^-ILK and total ILK in either HL-60 or THP-1 cells (Figure 2A). This suggested that T315 induced cytotoxicity of AML cells through an ILK-independent manner. Although the expression of Akt did not change (Figure 2B), cells treated with T315 exhibited down regulation of both p^Thr308^-Akt and p^Ser473^-Akt which was in contrast with the effect of T315 on prostate and breast cancer cells [2]. There was no change in protein expression of extracellular signal–regulated kinase 1 and 2 (ERK1/2) and phosphorylated ERK1/2 after T315 treatment.

### 2.3. T315 Induces Apoptosis, Caspase Activation and *Poly-ADP-Ribose Polymerase* (PARP) Cleavage in AML Cell Lines

**Figure 3:**
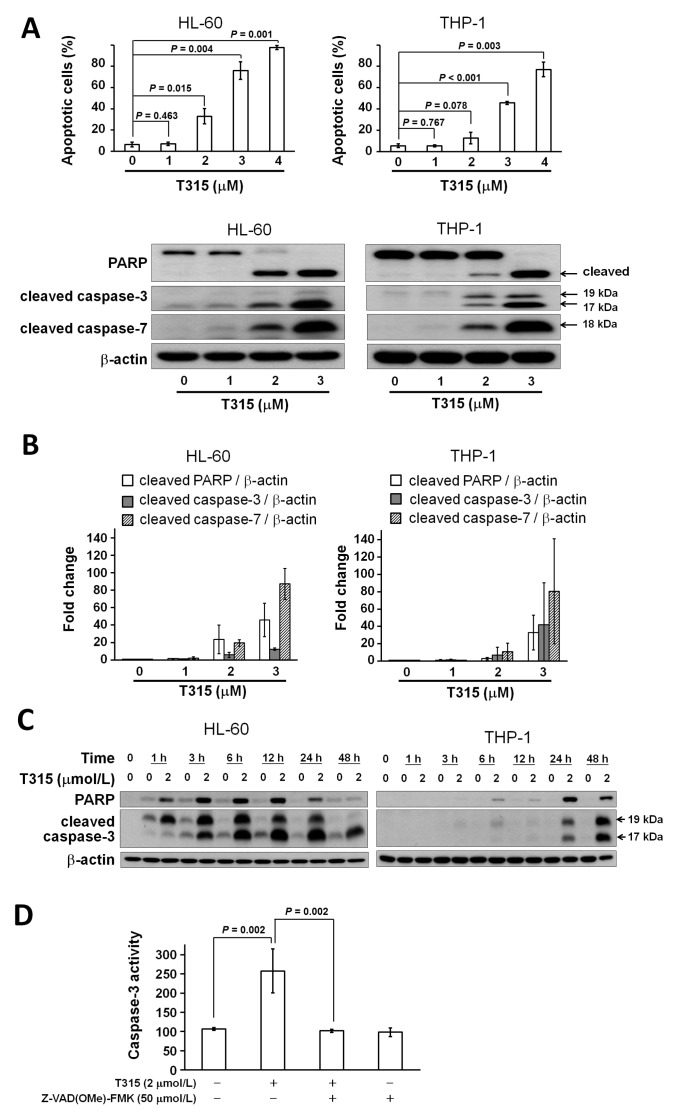
T315-mediated cytotoxicity is dependent on caspase activation and apoptosis. (**A**) T315 induced poly-ADP-ribose polymerase (PARP) cleavage and activation of caspase-3 and caspase-7 in HL-60 and THP-1 cells at 24 h. Protein extract of 20 µg from cell lysates were used for Western blot analysis; (**B**) Fold change of cleaved PARP/β-actin, cleaved caspase-3/β-actin, and cleaved caspase-7/β-actin in treatment with T315 of 1, 2 or 3 µM compared with DMSO control (*n* = 3); (**C**) Time course change of PARP cleavage and caspase-3 activation induced by T315 of 2 µM or DMSO control; (**D**) The increased caspase-3 activity in HL-60 cells treated with T315 for 24 h was rescued by pretreatment of 50 µmol/L Z-Val-Ala-Asp(OMe)-fluoromethyl ketone (Z-VAD(OMe)-FMK).

In order to determine if PARP cleavage and caspase activation occur in T315-mediated cytotoxicity, HL-60 and THP-1 cells were incubated with T315 at 0, 1, 2 or 3 µmol/L for 24 h. Western blotting showed that T315 induced PARP cleavage and caspase-3 and caspase-7 activation in HL-60 and THP-1 cell lines in a dose-dependent manner (Figure 3A). The histogram of cleaved PARP versus β-actin, cleaved caspase-3 versus β-actin, and cleaved caspase-7 versus β-actin change folds are shown in Figure 3B (*n* = 3). The time course of PARP cleavage and caspase-3 activation induced by T315 is shown in Figure 3C.

In order to further validate the caspase-3 activation induced by T315, HL-60 cells were incubated with T315 for 24 h with or without pretreatment of 50 µmol/L Z-Val-Ala-Asp(OMe)-fluoromethyl ketone (Z-VAD(OMe)-FMK), a pan-caspase inhibitor (Figure 3D). The increased caspase-3 activity was completely prevented by Z-VAD(OMe)-FMK treatment.

### 2.4. T315 Induces Autophagic Cell Death in AML Cell Lines

**Figure 4:**
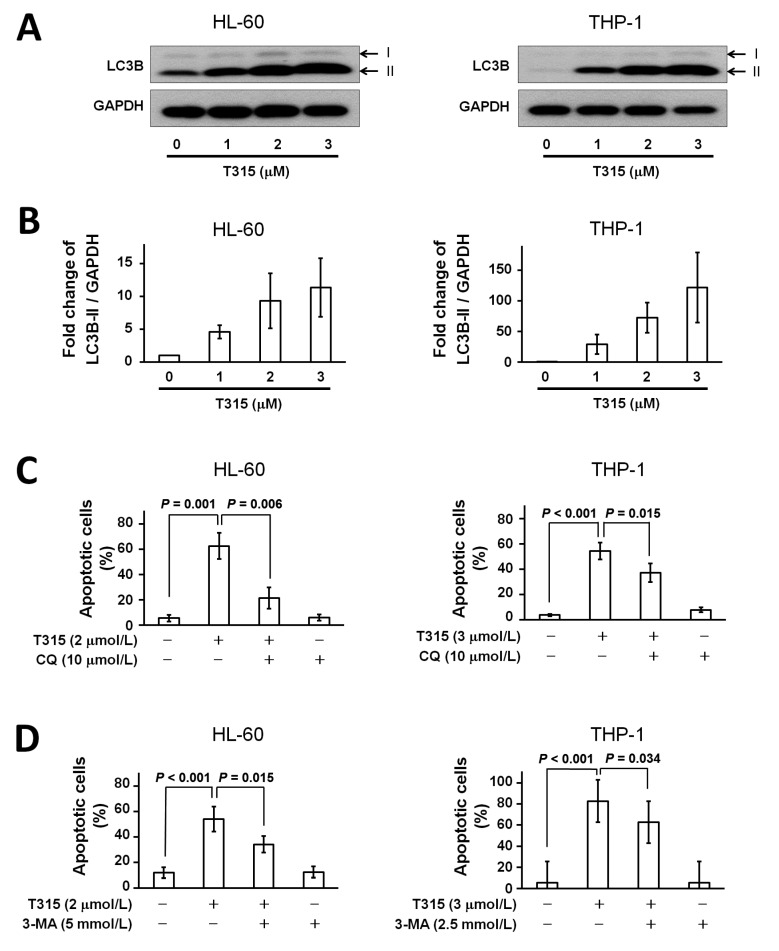

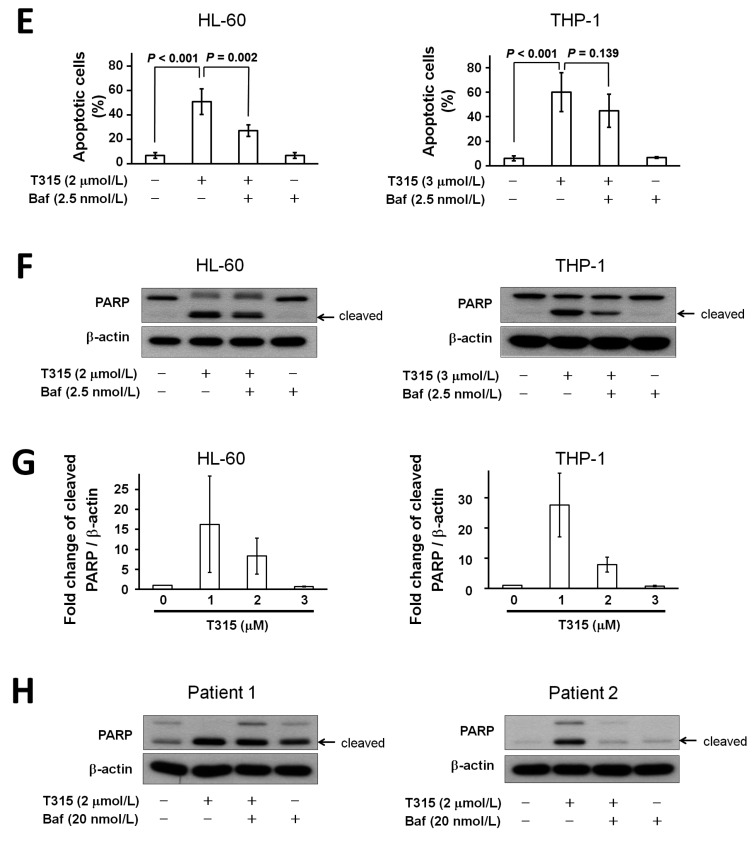
T315 induces autophagic cell death but not protective autophagy in AML cells. (**A**) T315 induced upregulation of LC3B-II in HL-60 and THP-1 cells. Cells (0.25 × 10^6^ cells/mL) were treated with indicated concentrations of T315 for 24 h. 20 µg protein from cell lysates were used for Western blot analysis; (**B**) Histogram of fold change of LC3B-II/GAPDH protein expression in cells treated with T315 for 24 h (*n* = 3); (**C**) T315-induced apoptosis was partially rescued by chloroquine (CQ), an autophagy inhibitor. Cells were treated with DMSO vehicle or T315 for 24 h with or without pretreatment of CQ for 1 h, and then analyzed by a flow cytometer. (*n* = 3 for HL-60 and *n* = 4 for THP-1 cells); (**D**) T315-induced apoptosis was partially rescued by 3-methyladenosine (3-MA), an autophagy inhibitor. Cells were treated with DMSO vehicle or T315 for 24 h with or without pretreatment of 3-MA for 1 h, and then analyzed by a flow cytometer. (*n* = 4 for HL-60 and n = 5 for THP-1 cells); (**E**) T315-induced apoptosis was partially rescued by bafilomycin-A1 (Baf), an autophagy inhibitor. Cells were treated with DMSO vehicle or T315 for 24 h with or without pretreatment of Baf for 1 h, and then analyzed by a flow cytometer. (*n* = 5 for HL-60 and *n* = 5 for THP-1 cells); (**F**) T315-induced PARP cleavage was partially rescued by Baf. Cells were treated with DMSO vehicle or T315 for 24 h with or without pretreatment of Baf for 1 h, and then analyzed by Western blotting; (**G**) Histogram of fold change of cleaved PARP/β-actin protein expression in cells treated with T315 with or without pretreatment of Baf for 1 h (*n* = 3); (**H**) T315-induced PARP cleavage in primary AML cells was partially rescued by Baf. Primary AML cells were treated with DMSO vehicle or T315 for 24 h with or without pretreatment of Baf for 1 h, and then analyzed by Western blotting (two patients’ data shown here).

Autophagy is a physiological process in which cellular components are degraded by lysosomal activity. Either autophagic cytoprotection or autophagic cell death has been shown to be important for the antileukemic effect of different chemotherapeutic agents [6]. Therefore, in addition to apoptosis, we investigated if autophagy was involved in T315-mediated cytotoxicity. Treatment with T315 for 24 h induced dose-dependent increases in microtubule-associated protein 1A/1B light chains 3B (LC3B)-II expression in HL-60 and THP-1 cells (Figure 4A). For comparison, histograms of fold changes of LC3B-II/glyceraldehyde 3-phosphate dehydrogenase (GAPDH) protein expression are shown in Figure 4B.

Next, to see if autophagic cell death contributed to T315-mediated cytotoxicity, HL-60 and THP-1 cells were treated with dimethyl sulfoxide (DMSO) vehicle control or T315 for 24 h with or without pretreatment of 3 kinds of autophagy inhibitors, chloroquine (CQ), 3-methyladenosine (3-MA), and bafilomycin-A1, and then analyzed for apoptosis (Figure 4C–E). Although the degree of apoptosis rescue varied, all 3 autophagy inhibitors lessened the cell apoptosis induced by T315. These findings implied that autophagic cell death contributed to T315-mediated cell apoptosis.

Compatible with the autophagy inhibitor-mediated rescue of cell apoptosis in flow cytometric analysis, pretreatment with bafilomycin-A1 for 1 h also lessened the PARP cleavage in AML cell lines (Figure 4F,G) and, more important, in primary AML cells (Figure 4H). In summary, T315 induced autophagic cell death, not protective autophagy, in AML cells.

### 2.5. T315-Mediated Cytotoxicity Is Rescued by Combination of an Apoptosis Inhibitor and an Autophagy Inhibitor

**Figure 5:**
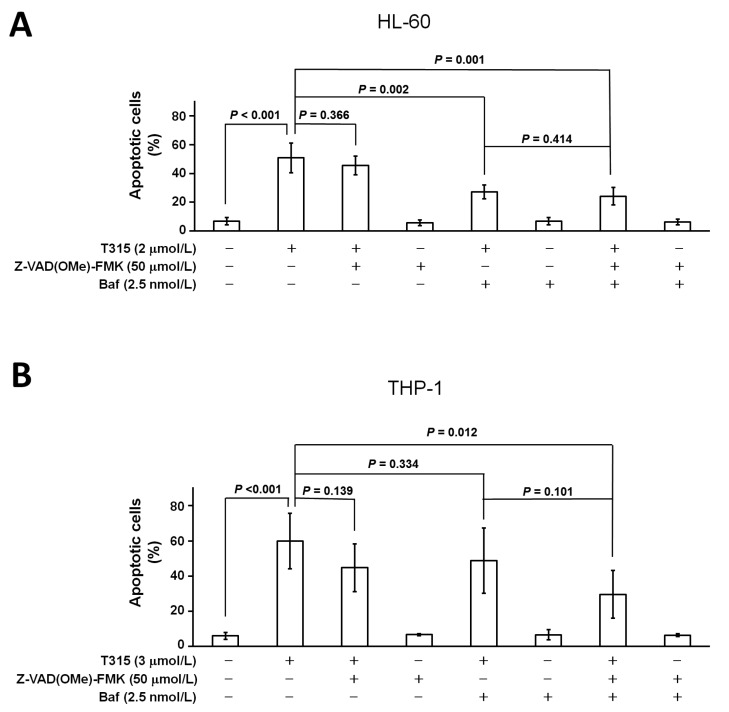
T315-mediated cytotoxicity is rescued by combination of an apoptosis inhibitor and an autophagy inhibitor. Cells were treated with DMSO vehicle or T315 for 24 h with or without pretreatment of Z-VAD(OMe)-FMK and/or bafilomycin-A1 (Baf) for 1 h, and then analyzed by a flow cytometer. (**A**) For HL-60 cells (*n* = 5); (**B**) For THP-1 cells (*n* = 5).

In light of the generation of both apoptosis and autophagic cell death, we further examined the combinatorial effect of an apoptosis inhibitor and an autophagy inhibitor on cell death induced by T315 (Figure 5A,B). For HL-60 cells, the combination of Z-VAD(OMe)-FMK and bafilomycin-A1 rescued more cells than Z-VAD(OMe)-FMK alone (*p* = 0.001). However, the difference of apoptosis rescued between Z-VAD(OMe)-FMK plus bafilomycin-A1 treatment and bafilomycin-A1 alone were less significant (*p* = 0.414). This suggests that autophagic cell death plays a more important role than apoptosis in T315-mediated death of HL-60 cells.

### 2.6. T315 Slows the Growth of THP-1 Xenografts and Prolongs the Survival of Tumor-Bearing Athymic Nude Mice

**Figure 6:**
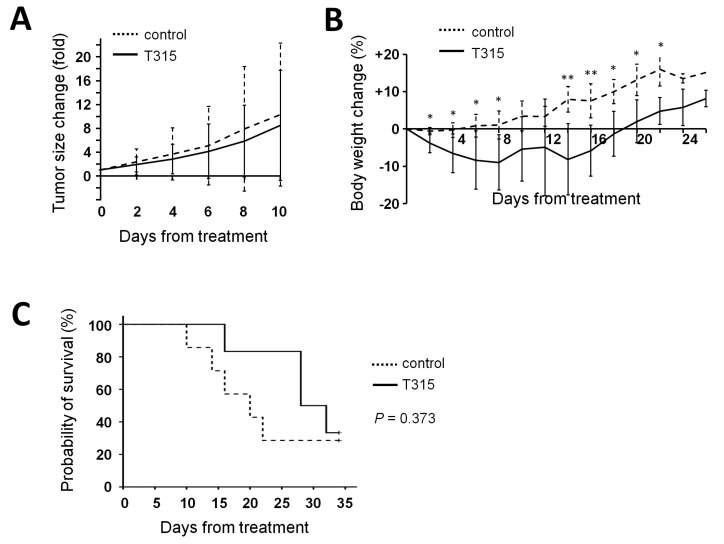
T315 mitigates the growth of THP-1 xenografts and prolongs the survival of tumor-bearing athymic nude mice. (**A**) Mice bearing THP-1 xenografts were treated with DMSO vehicle (······, *n* = 7) or T315 (**――**, *n* = 6) at 37.5 mg/kg/day intraperitoneally. The data represent group means and were plotted until day 10 when one mouse in the control group reached the endpoint tumor size (≥2000 mm^3^) and was sacrificed; (**B**) Body weight change of mice. The data were plotted until day 26 when the control group and treated group had 2 and 3 mice remaining, respectively; (**C**) Overall survival curve plotted by Kaplan-Meier method. * denotes *p* < 0.05; ** denotes *p* < 0.01 compared to the control group.

To investigate the anti-leukemia effect of T315 in vivo, thirteen male athymic nude mice were xenografted with THP-1 cells. Six mice in the treatment group received T315 intraperitoneally at a dose of 37.5 mg/kg per day, and seven mice in the placebo-control group received the DMSO vehicle daily. T315 had a trend to delay the growth of xenograft tumors (Figure 6A). Although mice in the T315 group had less body weight compared with those in the placebo-controlled group in the first days after initiation of treatment, the loss of body weight did not exceed the 20% endpoint criterion (Figure 6B). In terms of survival time, five mice in the placebo-treated and 4 mice in the T315-treated group reached the humane sacrifice criterion of tumor size (≥2000 mm^3^). Although most mice were sacrificed early due to tumor size, a T315-mediated delay in tumor growth was still evident. T315 prolonged the tumor-defined survival time by approximately eight days compared with controls, with median survival times of 20.0 ± 5.2 days and 28.0 ± 6.5 days in placebo control mice and T315 treated mice, respectively (Figure 6C, *p* = 0.373).

## 3. Discussion

We have described here the anticancer activity of an ILK inhibitor, T315, in both AML cell lines and primary AML cells. The T315-mediated decrease in cell viability is through both apoptosis and autophagic cell death. Akt and p308-Akt are also down-regulated. In addition, the tumor inhibitory effect of T315 is demonstrated in a THP-1 xenograft mouse model.

Autophagy is a cellular process in which intracellular components are engulfed, digested and recycled via the formation of autophagosomes and autolysosomes, important for cell survival under stress and harmful conditions [6]. This anti-apoptosis function of autophagy has important biological and pathological implications including ischemic injury, cancer therapy and chemoresistance [7]. In the context of cancer, this protective role of autophagy may actually promote tumor survival in a cellular environment of inadequate nutrition or during therapy. Over periods of prolonged stress or poor nutrition, however, autophagy may signal cell death by apoptosis when a cell can no longer survive by recycling organelles. Therefore, the process of autophagy is a double-edged sword in cancer and can either facilitate cancer cell survival or promote cell death depending on other internal and exernal stimuli [8,9].

Cell death mediated by autophagy, referred to as autophagic cell death or type II cell death, has been induced by cancer therapies and was thought to contribute to the death of leukemia [10,11], malignant glioma [12] and lung cancer cells [13]. Indeed, methods used to interfere with the autophagic cell death in these studies rescued the treated cells. Our study shows that T315 induces autophagic cell death but not protective autophagy in AML cells.

Regarding the induction of both apoptosis and autophagic cell death in the present study, it is interesting to note that pretreatment with different autophagy inhibitor rescues both HL-60 and THP-1 cells from apoptosis. This observation implies crosstalk between these modes of cell death rather than 2 independent pathways in AML cells (Figure 4). However, the interplay of apoptosis and autophagic cell death remains undefined.

Various studies have demonstrated an overlap in the regulatory machinery for apoptosis and autophagic cell death. Beclin 1, for example is a protein required for autophagy and also belongs to an apoptosis-requlating domain of proteins. Stressed cells can undergo autophagy induced by Beclin 1 or can undergo apoptosis [8]. Caspase-mediated Beclin 1 cleavage and Beclin 1-Bcl-2 interaction are 2 examples of nodes of crosstalk between autophagy and apoptosis reviewed by Su et al. [9]. One of the most studied and characterized molecular regulator of autophagy and apoptosis is p53 localization [7]. It has been reported that cytoplasmic p53 inhibits autophagy and induces apoptosis while nuclear localization of p53 stimulates both apoptosis and autophagy via the transactivation of target genes [14,15]. In our experiment, the rescue of cell apoptosis by autophagy inhibitors also suggests a crosstalk between autophagy and apoptosis (Figure 4). Our data provides evidence that autophagic cell death and apoptosis can act cooperatively to achieve a cell killing effect. Further mechanistic studies are needed to better characterize the crosstalk between autophagy and apoptosis in AML.

Although our results show a convincing antileukemia effect of T315 in vitro and in vivo, some limitations of our study are noteworthy. First, while the combination of Z-VAD(OMe)-FMK and an autophagy inhibitor resulted in greater rescue from apoptosis induced by T315, the rescue was not complete. This partial rescue of apoptosis in AML cells by both Z-VAD(OMe)-FMK and autophagy inhibitors suggests the existence of other mechanisms of T315-mediated cell death in AML cells. Second, even though T315 delayed the growth of THP-1 xenografts compared to the vehicle control group, most animals in both groups were sacrificed early due to the tumor size reaching the pre-set size criterion. Further modification of T315 to improve the efficacy and to reduce the toxicity is necessary for clinical application.

In conclusion, T315 exhibits a potent antileukemia effect in both AML cell lines and primary AML cells with cell death mediated, at least in part, through generation of apoptosis and autophagic cell death. In vivo, T315 inhibits AML xenograft tumor growth. Collectively, this study provides additional clarity to the anticancer activity of T315 that will be useful in furthering its development for the treatment of AML and possibly other hematological malignancies.

## 4. Materials and Methods

### 4.1. Cells and Culture Conditions

Primary AML cells were isolated from freshly collected bone marrow using Ficoll-Paque^TM^ PLUS (GE Healthcare Bio-Sciences AB, Uppsala, Sweden) according to the manufacturer’s instructions if the leukemia cells accounted for more than 90% of non-erythroid mononucleated cells of bone marrow. Normal bone marrow nucleated cells were harvested using Ficoll-Paque^TM^ PLUS from patients with treatment-naive non-Hodgkin’s lymphoma for whom bone marrow examination for lymphoma staging was performed but determined to be normal. All bone marrow samples were obtained under a protocol approved by the China Medical University Hospital internal review board (CMUH102-REC1-124 issued on 26 May 2014). Written informed consent was obtained from all patients in accordance with the Declaration of Helsinki. Human AML cell lines HL-60 (ATCC CCL-240) and THP-1 (ATCC TIB-202) were from American Type Culture Collection (ATCC, Manassas, VA, USA). All cells were incubated in RPMI-1640 media (Invitrogen, Carlsbad, CA, USA) supplemented with 10% heat-inactivated fetal bovine serum (FBS; Invitrogen) and penicillin (100 U/mL)/streptomycin (100 µg/mL) (Invitrogen) at 37 °C in the presence of 5% CO_2_.

### 4.2. Reagents

T315 {*N*-methyl-3-(1-(4-(piperazin-1-yl)phenyl)-5-(4′-(trifluoromethyl)-[1,1′-biphenyl]-4-yl)-1*H*-pyrazol-3-yl)propanamide} was synthesized as previously described [2], with identity and purity (≥99%) verified by proton nuclear magnetic resonance, high-resolution mass spectrometry, and elemental analysis. For in vitro experiments, T315 was dissolved in dimethyl sulfoxide (DMSO), and added to the culture medium with a final DMSO concentration less than 0.1%. The pharmacological agents were purchased from the respective vendors: bafilomycin-A1 (Cayman Chemical, Ann Arbor, MI, USA); chloroquine (Sigma-Aldrich, St. Louis, MO, USA); 3-methyladenine (3-MA; Sigma-Aldrich); Z-VAD(OMe)-FMK (Santa Cruz Biotechnology, Santa Cruz, CA, USA).

### 4.3. MTS Assay

Measurement of cell growth was performed using CellTiter 96 Aqueous Non-radioactive Cell Proliferation Assay kit purchased from Promega (Madison, WI, USA). Cells (0.25 × 10^6^/mL) were placed in 200 µL volume in 96-well microtiter plates with the indicated reagent and incubated at 37 °C [16]. MTS solution [3-(4,5-dimethylthiazol-2-yl)-5-(3-carboxymethoxyphenyl)-2-(4-sulfophenyl)-2*H*-tetrazolium] and PMS (phenazine methosulfate) solution were mixed 20:1 by volume. The colorimetric measurements were performed 4 h later at 490-nm wavelength by a VersaMax tunable microplate reader (Molecular Devices, Sunnyvale, CA, USA). The cell viability was expressed as a percentage of absorbance value in treated samples compared to that observed in control vehicle-treated samples (subtract the blank in both conditions).

### 4.4. Cell Viability and Apoptosis Assay by Flow Cytometry

Cell viability was assessed by dual staining with annexin V conjugated to fluorescein isothiocyanate (FITC) and propidium iodide (PI) [17]. Cells (0.5 × 10^6^) were stained by annexin V-FITC (BD Pharmingen, San Diego, CA, USA) and PI (BD Pharmingen) according to the manufacturer’s instructions. Cells were analyzed by a flow cytometer BD FACSCanto II (BD, Franklin Lakes, NJ, USA). Viable cells were those with both annexin V-FITC negative and PI negative staining. The viable cells in each sample were expressed as % by normalizing annexin V-/PI- cells to control. Annexin V-FITC positive cells were identified as apoptotic cells [18].

### 4.5. Western Blotting

Cell lysates were prepared using RIPA buffer (150 mmol/L NaCl, 50 mmol/L Tris pH 8.0, 1% NP40, 0.5% sodium deoxycholate and 0.1% sodium dodecyl sulfate) supplemented with protease inhibitor (Sigma-Aldrich) and phosphatase inhibitor cocktail (Calbiochem, Darmstadt, Germany) [19]. Antibodies against various proteins were obtained from the following sources: poly-ADP-ribose polymerase (PARP), p^Thr308^-Akt, p^Ser473^-Akt, cleaved caspase-3, LC3B, cleaved caspase-7 (Cell Signaling, Danvers, MA, USA); Akt, ERK1/2, p^Thr202Tyr204^-ERK1/2, GAPDH, ILK, p^Thr173^-ILK (Santa Cruz Biotechnology); β-actin (Sigma-Aldrich). The goat anti-rabbit IgG-horseradish peroxidase (HRP) conjugates and goat anti-mouse IgG-HRP conjugates were purchased from Jackson ImmunoResearch Laboratories, Inc. (West Grove, PA, USA).

### 4.6. Analysis of Caspase-3 Activity

Caspase-3 activity was assessed using a FITC rabbit anti-active caspase-3 kit (BD Pharmingen) according the manufacturer’s protocol.

### 4.7. In Vivo Therapeutic Efficacy Evaluation of T315 in the THP-1 Xenograft Model

The in vivo efficacy evaluation of T315 was carried out using a xenograft model in athymic nude mice [16]. Thirteen male nude mice of 5 to 7 weeks of age were obtained from the National Laboratory Animal Center (Taipei, Taiwan). The mice were housed under conditions of constant photoperiod (12 h light and 12 h dark) with ad libitum access to sterilized food and water. THP-1 cells were cultured in RPMI-1640 supplemented with 10% heat-inactivated FBS. Before inoculation, THP-1 cells were washed with PBS twice and resuspended in a mixture of RPMI-1640 and Matrigel (BD Matrigel^TM^ Basement Membrane Matrix; BD) with a 1:1 volume ratio. Each mouse was inoculated over the flank subcutaneously with 1 × 10^7^ THP-1 cells in a total volume of 0.2 mL. Tumor diameter was measured every three days using calipers and the tumor volume was calculated using a standard formula: width^2^ × length × 0.52. Body weights of the mice were measured every three days. When the mean tumor volume had reached 50 mm^3^, mice were randomized to two groups (seven mice and six mice in placebo-control group and treatment group, respectively). The mice in the treatment group received T315 (concentration 18.75 mg/mL = 35.14 mmol/L) intraperitoneally once daily at a dose of 37.5 mg/kg per day (for example, volume injected is 50 µL for a mouse weighting 25 g), and the mice in the placebo-control group received the DMSO vehicle. All mice received treatments daily until reaching the endpoint. Humane endpoint criteria included body weight loss more than 20% or tumor size more than 2000 mm^3^. Scheduled terminal sacrifice for surviving mice occurred on day 35 after initiation of T315 or placebo. The in vivo experiment protocol was approved by the Institutional Animal Care and Use Committee of China Medical University (Taichung, Taiwan, IACUC Approval no.: 104-87-N, period of protocol valid from 1 August 2015 to 31 July 2017).

### 4.8. Statistical Analysis

Nonlinear mixed models were used to obtain IC_50_. Two-tailed unpaired *t-*test was used for comparisons of two sets of data. Kaplan-Meier overall survival curve of mice was analyzed using log rank test. All statistical analysis was performed with SPSS for Windows (SPSS, Inc., Chicago, IL, USA).

## Abbreviations

AML	acute myeloid leukemia
DMSO	dimethyl sulfoxide
FITC	fluorescein isothiocyanate
HRP	horseradish peroxidase
ILK	integrin-linked kinase
PARP	poly-ADP-ribose polymerase
PI	propidium iodide
